# Undocumented migrants lack access to pregnancy care and prevention

**DOI:** 10.1186/1471-2458-8-93

**Published:** 2008-03-19

**Authors:** Hans Wolff, Manuella Epiney, Ana P Lourenco, Michael C Costanza, Jacqueline Delieutraz-Marchand, Nicole Andreoli, Jean-Bernard Dubuisson, Jean-Michel Gaspoz, Olivier Irion

**Affiliations:** 1 Department of Community Medicine and Primary Care, Geneva University Hospitals, University of Geneva, Geneva, Switzerland; 2 Department of Obstetrics and Gynaecology, Geneva University Hospitals, University of Geneva, Geneva, Switzerland; 3 Unit of Populational Epidemiology, Geneva University Hospitals, University of Geneva, Geneva, Switzerland

## Abstract

**Background:**

Illegal migration is an increasing problem worldwide and the so-called undocumented migrants encounter major problems in access to prevention and health care. The objective of the study was to compare the use of preventive measures and pregnancy care of undocumented pregnant migrants with those of women from the general population of Geneva, Switzerland.

**Methods:**

Prospective cohort study including pregnant undocumented migrants presenting to the University hospital from February 2005 to October 2006. The control group consisted of a systematic sample of pregnant women with legal residency permit wishing to deliver at the same public hospital during the same time period.

**Results:**

161 undocumented and 233 control women were included in the study. Mean ages were 29.4 y (SD 5.8) and 31.1 y (SD 4.8) (p < 0.02), respectively. 61% of undocumented women (controls 9%) were unaware of emergency contraception (OR 15.7 (8.8;28.2) and 75% of their pregnancies were unintended (controls 21%; OR 8.0 (4.7;13.5)). Undocumented women consulted for an initial pregnancy visit more than 4 weeks later than controls and only 63% had their first visit during the first trimester (controls 96%, p < 0.001); 18% had never or more than 3 years ago a cervical smear test (controls 2%, OR 5.7 (2.0;16.5)). Lifetime exposure to violence was similar in both groups, but undocumented migrants were more exposed during their pregnancy (11% vs 1%, OR 8.6 (2.4;30.6)). Complications during pregnancy, delivery and post-partum were similar in both groups.

**Conclusion:**

Compared to women who are legal residents of Geneva, undocumented migrants have more unintended pregnancies and delayed prenatal care, use fewer preventive measures and are exposed to more violence during pregnancy. Not having a legal residency permit therefore suggests a particular vulnerability for pregnant women. This study underscores the need for better access to prenatal care and routine screening for violence exposure during pregnancy for undocumented migrants. Furthermore, health care systems should provide language- and culturally-appropriate education on contraception, family planning and cervical cancer screening.

## Background

An estimated 8'000 to 12'000 undocumented migrants live and work in the canton of Geneva, representing 1.8 to 2.9% of the 440'982 resident population [1,2]. These so-called undocumented migrants live in Geneva *without* a legally mandatory residence permit. Undocumented migrants arrive in Switzerland in general as tourists but do not leave Switzerland, which ordinary tourists are required to do after a maximum stay of three months. The restrictive Swiss immigration policy makes it almost impossible for low-qualified migrants from countries outside the European Union to receive a legal residency permit. Because of difficult living conditions, separation from their families, frequent exploitation by employers, permanent threat of being caught by the police, and exclusion from the usual health care system, undocumented migrants are at increased risk of poor health. Pregnancy can be of particular concern, as it may imply loss of work and of income just when these resources are sorely needed to pay for medical care.

However, there is a striking scarcity of direct evidence on these issues and study results are controversial. Some [3-5], but not all studies show that lack of health insurance, illegal status, and low income increase maternal and newborn morbidity. On the other hand, a "healthy migrant effect" has been postulated to explain the lower prevalence of low-birthweight newborns in foreign-born compared to US-born women [6]. In a previous study on pregnant, undocumented migrant women in Geneva, we found a high percentage of unintended pregnancies (83%), and a low use of important preventive measures, such as rubella immunisation or cervical cancer screening; however, comparison with the general population was difficult because control group data were not available [7].

The objective of the present study was to describe detailed information about contraception, intendedness of pregnancy, health status and behaviour, violence exposure, and birth outcomes of undocumented migrant women and to compare them to those of women having a legal residence permit.

## Methods

### Setting

In Switzerland, health care insurance is legally mandatory and every legal resident has to arrange for coverage on their own. However, as the cost of health care insurance is high, more than 90% of Geneva's undocumented migrants in Geneva lack such insurance. Since 1996, a health care unit has offered medical care for free or at low cost to undocumented migrants in Geneva, where no similar site for free gynaecological or general health care exists. Over the years undocumented migrants have learned to trust the unit which does not transmit information to the police or other official agencies. The consultations in general medicine of the facility reached 10'000 in 2006 for over 3'000 undocumented migrants. Because of its high visibility for the local undocumented population and of the absence of formal administrative requirements, the unit reached the majority of pregnant, undocumented and uninsured women [8,9].

### Study plan and population

#### Pregnant, undocumented migrants (exposed group)

This prospective cohort study included all pregnant, undocumented women presenting to the health care facility between February 2005 and October 2006 as the exposed group. All pregnant women wishing to deliver were included independently of their stage of pregnancy and systematically referred to a coordinating midwife providing free pregnancy care in collaboration with the women's University Hospital, which is the only public women's hospital in Geneva. Some pregnant undocumented women who presented directly to that hospital were also referred to the same midwife. The women's hospital is well known and frequented by the majority of the female population of Geneva. In 2006, 67% of the 5'892 newborn of Geneva were born in this hospital.

#### Control group

The control group comprised a systematic sample of women *with* a legal residency permit who were directed by their gynaecologist to the antenatal consultation at the women's hospital. They were seen by the same midwife as undocumented migrants. These women were selected during predetermined days between November 2005 and May 2006. On each selected day, every woman who saw the midwife was asked to participate in the study.

#### Exclusion criteria

Women who were unable to provide informed consent were excluded form the study as well as women who decided to have voluntary termination of pregnancy (TOP). No women met the first criterion but 175 undocumented migrants were excluded because of voluntary TOP.

### Questionnaire and blood tests

A socio-demographic questionnaire was completed during a face-to-face interview by a fluently Spanish and French-speaking midwife for both groups. The data were collected during pregnancy follow-up (first to third trimester) for undocumented migrants and during the last trimester for control women. The socio-demographic questionnaire included 31 items concerning health insurance, nationality, children, housing and working conditions, duration and aim of the stay in Geneva, education, occupation, social support, major difficulties in daily living and social support, which was evaluated by: civil status, presence of a family member in Geneva, and relation of the father with the child. The health questionnaire concerned contraceptive history, health problems during pregnancy and post-partum as well as intendedness of pregnancy, meaning that pregnancies are begun without planning or intent. Cervical and breast cancer screening histories and substance abuse were also assessed. Prenatal aneuploidy screening, cytomegalovirus (CMV), venereal disease research laboratory test (VDRL), human immunodeficiency virus (HIV) Hepatitis B, as well as rubella and Toxoplasma gondii immunity status were assessed by blood tests. Information concerning delivery and neonatal outcomes was obtained from medical records.

### Main outcomes and potential confounders

The main study outcomes were unintendedness of pregnancy, knowledge of the emergency pill, cervical and breast cancer screening, delayed prenatal care, violence exposure during pregnancy and complications during pregnancy, delivery and post-partum. As the main confounding factors we considered age, Latin American origin, civil status, education, duration of residence in Geneva, and having emotional support and/or a family member in Geneva.

### Statistical analysis

In order to investigate the relationship between legal status and the main outcomes we first used 2 × 2 tables and performed Chi-square and Fisher's exact tests to compare proportions for categorical variables and unpaired student's t-tests to compare means for continuous variables. Then we performed the same analyses after stratifying for the potential confounding factors. Finally we used multiple logistic regression analysis adjusting systematically for age and those confounding factors that remained statistically significant (p < 0.05) in the models. Women who left the country before delivery were excluded from analyses concerning complications, delivery and post-partum. All analyses were performed using SPSS for Windows (version 15.0).

### Ethical considerations

The study was approved by the Geneva University Hospitals Ethics Committee (1.2.2005, no 05058-CD). All participating undocumented migrant and control women were informed that their responses would be treated anonymously and provided written consent.

## Results

During the 20 month study period, 163 undocumented women wishing to deliver were referred to the midwife. None had valid health insurance at time of their first pregnancy visit. Two-hundred-forty-six women with a regular residency permit were selected to participate in the control group. Two undocumented women (age unknown) and 13 controls (mean age 28.5) refused participation for the following reasons: had no time (n = 6); did not want to participate without giving a reason (n = 4); husband didn't want (n = 2); didn't want to reply to questions (n = 2); was afraid (n = 1). Thus, the final study group consisted of 161 undocumented women and 233 women with a legal residency permit.

### Missing data and losses to follow-up

Socio-demographic data were missing in up to 39 undocumented migrants (24.2%) compared to 3 controls (1.3%) and intentional pregnancy status was missing in 12 undocumented women (7.5%) and in no controls. Knowledge of the emergency contraceptive pill was missing in 40 undocumented migrants (24.8%); otherwise preventive aspects, history of voluntary abortion and of violence, or blood tests were missing in less than 10% of all participants. Delivery, post-partum and neonatal information were missing in 54 undocumented women (33.5%) who left Geneva and were considered as lost to follow-up (controls 3 (1.3%)). When comparing undocumented women who delivered at the women's hospital to those lost to follow-up no statistical difference was found concerning: weeks of pregnancy at first consultation, alcohol, tobacco or drug consumption, depression, social support, violence exposure and knowledge, and use of preventive measures. One woman in each group had a late spontaneous abortion (respective weeks 16 and 17), hence were excluded from the delivery, post-partum, and neonatal outcome analyses.

### Nationality, reasons for migration, civil status, and social support

The large majority (84%) of undocumented migrants came form Latin America. Reported reasons for migration were mainly economical (84%), but also were related to family (7%) (to join a husband or a relative), education (2%, to study French), and 7% for political or tourism reasons.

Fifteen percent of the undocumented pregnant migrants indicated lack of emotional support (controls 0.4%, p < 0.001). Furthermore they reported less social support than controls: 71% were single (controls 21%, p < 0.001); 18% lived separately from the father of their future child (controls 4%, p < 0.001); and 8% had no relationship with the father (controls 0.4%, p < 0.001). Twenty two percent of the undocumented migrants had no or only an occasional relationship with the father of their child (controls 1.3%, p < 0.001). Forty-three percent of the undocumented women with children lived separately from them (controls 1%, p < 0.001). Moreover, only 47% of undocumented women had a family member living in Geneva (controls 68%, p < 0.001).

### Housing, education, and occupation

Sixty percent of undocumented women (controls 18%) shared a single room with on average two (controls one) other persons. Over two-thirds (70%) of the undocumented were employed, mostly (95%) in the domestic sector (childcare and house keeping). They worked a mean of 23.9 hours a week (SD 15.8) and earned 13 SFr (SD 8.4; ≈8 Euro) per hour, which is 40% lower than the minimal mandatory hourly wage in Geneva.

### Main difficulties in life

To the open-ended question "which is actually the most important difficulty in your life?", 46% of the undocumented migrant women answered illegality, 25% housing, 14% finding work, 9% the language (French), 5% absence of the family, and 2% other. The control women reported: housing (22.9%), lack of money (22.9%), to find work (15.7%), and language (7.2%). Other sociodemographic characteristics are summarized in Table 1.

**Table 1:** Sociodemographic characteristics of undocumented pregnant migrants vs. pregnant women with legal residency status (control group) who delivered at the Geneva University Hospital, Switzerland between February 2005 and October 2006.

Characteristic or measurement	Undocumented (n = 161)	Missing n (%)	Control group (n = 233)	Missing n (%)	p-value
Mean age in years (SD)	29.4 (5.8)	-	31.1 (4.8)	-	0.02
Continent (%)	Latin America 83.9, Asia 6.2, Europe 5.6, Africa 4.3	-	Europe 80.3, Latin America 9.0, Africa 7.3, Asia 1.3, N.-Am. 2.1	-	< 0.001
Nationalities (%)	Bolivia 34.8, Brazil 23.0, Columbia 8.7, Equator 6.2, Peru 5.6, Philippines 3.7	-	Switzerland 49.4, Portuga 13.7, France 9.0, Spain 3.4, Brazil 3.0	-	< 0.001
Civil status (%)		-		-	< 0.001
Single	71.4%		20.6%		
Married	20.5%		72.9%		
Divorced	6.8%		5.2%		
Widowed	1.2%		1.3%		
Education:		39 (24.2)		3 (1.3)	
Years of schooling (SD)	12.7 (2.7)		13.5 (4.2)		0.07
Highest achieved education:					< 0.001
Primary school	3.7%		2.2%		
Secondary school	31.5%		47.4%		
High school degree	40.7%		15.2%		
University	24.1%		35.2%		
Years living in Geneva (SD)	2.5 (2.2)	39 (24.2)	16.4 (11.8)	18 (7.7)	< 0.001
Father not living in Geneva	18.2%	39 (24.2)	4.3%	-	< 0.001
Having a family member in Geneva	46.7%	39 (24.2)	67.7%	1 (0.4)	< 0.001
Having children	41.8% (of whom 56.9% in Geneva)	39 (24.2)	52.8% (of whom 99% in Geneva)	-	0.05
Absence of emotional support	15.0%	39 (24.2)	0.4%	-	< 0.001
Living conditions:		39 (24.2)		-	
Living in a single-room (%)	62.5%		18.0%		< 0.001
Total number of persons of those living in a single room (SD)	3.1 (SD 1.8)		2.3 (SD 0.83)		0.01
Moves during the last year (SD)	1.5 (1.2)		0.4 (1.2)		< 0.001
Lack of health insurance	100%	-	0%	-	< 0.001
Insured during pregnancy follow up	25.2%	-	0%	-	< 0.001

### Intendedness of pregnancy and contraception

In undocumented women, unintended pregnancies were significantly more frequent and accounted for 75% compared to 21% in the controls (adjusted OR 8.0 (4.7;13.5)). Sixty-one percent of the undocumented migrants were unaware of emergency contraception (Levonorgestrel) compared to 9% among the control group (adjusted OR 15.7 (8.8;28.2)). Moreover, 79% of the undocumented women with unintended pregnancies did not use any (48%) or used unreliable (31%) contraceptive measures, such as condoms, retraction, or the temperature method. Reasons for absence of contraception among undocumented migrants were: infrequent intercourse (25%), believed that they were infertile (18%), and stopped contraception (run out of pills, side effects, lack of money) (12%). Other less frequent reasons were: "didn't think about it", "latent wish of pregnancy but planned for later", "lack of knowledge about contraceptive methods", and "presumed sterility of the partner". Undocumented migrants declared their pregnancies as unintended 73.2% of the time when asked during the first and 78.8% during the second and third trimesters (p = 0.45).

### Preventive measures and violence exposure

There was significant under-use of preventive measures such as cervical smear (Pap) test and breast examination by undocumented migrants (Tables 2 and 3): they had more than a six-fold higher risk of under-use of Pap-test screening (never or > 3 years ago) (adjusted OR 5.7 (2.0;16.5) and a ten-fold higher risk of never having had a breast examination by a physician (adjusted OR 9.6; CI:4.5;20.5). When considering alcohol, tobacco, and other substance abuse, undocumented migrants showed a healthier pattern compared to the control group. Lifetime exposure to violence was similar in both groups, but undocumented migrants were more exposed during their pregnancy (11% vs 1%, adjusted OR 8.6 (2.4;30.6)).

**Table 2:** Preventive aspects and voluntary termination of pregnancy (TOP) history of undocumented pregnant migrants vs. pregnant women with legal residency status (control group) who delivered at the Geneva University Hospital, Switzerland between February 2005 and October 2006.

Characteristic or measurement	Undocumented (n = 161)	Missing n (%)	Control group (n = 233)	Missing n (%)	p-value
Unintendedness of pregnancy (%)	75.2	12 (7.5)	20.6	-	< 0.001
Of those with unintended pregnancies:					
No contraception	47.7%		33.3%		0.13
Insecure contraception (condom calendar, retraction)	31.2%		33.3%		0.51
No knowledge of emergency pill	61.2%	40 (24.8)	9.0%	-	< 0.001
Previous voluntary termination of pregnancy (TOP)	27.0%	13 (8.1)	24.0%	4 (1.7)	0.51
Pap-test:		15 (9.3)		-	
Never	13.0%		0%		< 0.001
> 3 years ago	4.8%		2.1%		0.15
Never breast examination by physician	29.7	16 (9.9)	3.9%	-	< 0.001
Tobacco never	65.5%	13 (8.1)	48.1%	-	0.001
Alcohol consumption:		16 (9.9)		-	
Before pregnancy	65.0%		73.8%		0.06
During pregnancy	11.7%		30.0%		< 0.001
Binge drinking (> 4 glasses/occasion):					
Before pregnancy	6.3%		15.5%		0.007
During pregnancy	4.2%		0.9%		0.03
Other substances abuse	3.4%	14 (8.7)	17.4%	3 (1.3)	
Exposure to **violence**:		16 (9.9)		2 (0.9)	
Lifetime	26.4%		32.2%		0.21
During pregnancy	11.2%		1.3%		< 0.001
Type of violence, if exposed to violence:					
Physical	53.7%		46.0%		0.45
Sexual	24.4%		28.6%		0.63
Psychological	53.7%		53.9%		0.98

**Table 3:** Unadjusted and adjusted relationships of pregnancy care or preventive aspects of undocumented migrants vs. women with legal residency status (control group) who delivered at the Geneva University Hospital, Switzerland between February 2005 and October 2006

Characteristic or measurement	Unadjusted OR (95% CI)	Adjusted OR (95% CI)
Unintended pregnancy	11.7 (7.2;19.0)	8.0 (4.7;13.5)°°
Delayed prenatal care (> 12 weeks of amenorrhea)	13.3 (6.3;27.9)	10.8 (4.8;24.2)°°
Violence during pregnancy	9.6 (2.7;33.5)	8.6 (2.4;30.6)°
Pap-test never or more than 3 years ago	9.9 (3.7;26.4)	5.7 (2.0;16.5)°°
Never breast examination by physician	10.5 (4.9;22.3)	9.6 (4.5;20.5)°
No knowledge of emergency pill	15.9 (8.9;28.3)	15.7 (8.8;28.2)°

° adjusted for age

°° adjusted for age and civil status

### Pregnancy characteristics

Undocumented migrants had an 11-fold higher risk for delayed prenatal care, meaning that their first pregnancy consultation occurred after the first trimester (adjusted OR 10.8 (CI 4.8;24.2)). Significant differences were also observed for Toxoplasma gondii and CMV immunity (Table 4).

**Table 4:** Pregnancy characteristics of undocumented migrants vs. women with legal residency status (control group) who delivered at the Geneva University Hospital, Switzerland between February 2005 and October 2006.

Characteristic or measurement	Undocumented (n = 161)	Missing (%)	Control group (n = 233)	Missing (%)	p-value
Weeks of pregnancy at first control (SD)	12.6 (6.1)	9 (5.6)	8.0 (3.1)	-	< 0.001
Trimester care began		9 (5.6)		-	< 0.001
First	63.2%		96.1%		< 0.001
Second	32.2%		3.4%		< 0.001
Third	4.6%		0.4%		0.007
No Toxoplasma gondii immunity	31.2%	9 (5.6)	57.9%	-	< 0.001
No Rubella immunity	10.0%	9 (5.6)	4.7%	-	0.046
No CMV immunity	7.8%	10 (6.2)	40.8%	49 (21)	< 0.001
VDRL positive	1.3%	10 (6.2)	0.4%	1 (0.4)	0.71
HIV positive	1 woman	10 (6.2)	1 woman	5 (2.1)	0.79
O'Sullivan test positive	7.8%	10 (6.2)	7.4%	17 (7.3)	0.96
HBV (Ag Hbs+)	0	9 (5.6)	0	-	
HCV	0.6%	10 (6.2)	0.4%	1 (0.4)	0.79
Prenatal screening (p.s.):		8 (5.0)		6 (2.6)	
Without p.s.	17.0%		6.2%		0.001
If p.s., double test (1. trimester):	54.6%		85.5%		< 0.001
Double test (2. trimester)	14.5%		7.5%		0.028
Amniocentesis	3.3%		9.7%		0.001

### Health problems at birth and complications

During the study period 106 undocumented women (66%) delivered at the women's hospital. Fifty four women left Geneva and one had an early abortion (week 16). Mean gestational age was lower in undocumented migrants and preterm births seemed to occur more frequently but lacked statistical significance (p = 0.09). Birth weight was similar in both groups. No significant differences between the exposed and control groups were found for complications during pregnancy, delivery, or post-partum. The main complications during pregnancy among undocumented migrants (controls) were: urinary infection 12% (11%), anaemia 8% (3%), risk of preterm delivery 7% (7%), vaginal bleeding 3% (4%), hypertension 3% (4%), and diabetes 2% (1%). Complications during vaginal delivery were similar in both groups: vaginal tear 11% (controls 18%), retention of the placenta 3% (4%), pre-eclampsia 2% (2%), and fever 1% (2%). Other birth outcomes are shown in Table 5.

**Table 5:** Birth outcomes and complications of undocumented migrants vs. women with legal residency status (control group) who delivered at the Geneva University Hospital, Switzerland between February 2005 and October 2006

Characteristic or measurement	Undocumented births (n = 106)	Missing n (%)	Control group (n = 229)	Missing n (%)	p-value
Gravidity (SD)	2.3 (1.4)	-	2.4 (1.4)	-	0.97
Parity (SD)	1.7 (1.0)	-	1.7 (0.9)	-	0.99
Mean gestational age (SD)	38.9 (1.9)	-	39.4 (1.4)	-	0.02
Pre-term births (< 37 weeks)	8.5%		3.9%		0.09
Post-term births (> 40 weeks)	15.1%	-	21.0%	-	0.20
Delivery:		-		-	0.14
Vaginal spontaneous	59.8%		63.9%		
Forceps	3.7%		4.3%		
Vacuum	11.2%		10.9%		
Cesarean	25.2%		20.0%		
Complications during pregnancy	31.1%	-	33.6%	-	0.70
Complications during delivery*	26.3%	-	32.8%	-	0.36
Complications during post partum	3.7%	-	2.2%	-	0.41
Sex of the child (male)	50.5%	-	54.6%	1 (0.4)	0.48
Health of the child		-		-	
Good health	96.2%		95.6%		0.53
Born dead	0.9%		0.4%		0.58
Transfer to Neonatology for serious health hazard)	3 (2.8%)		9 (3.9%)		0.44
Birth weight in g (SD)	3293.9 (521)	-	3380.9 (495)	-	0.15
Low birth weight (< 2500 g)	4.7%		2.6%		0.24
Apgar 1 (mean (SD))	8.5 (1.4)	-	8.7 (1.3)	1 (0.4)	0.43
Apgar 2 (mean (SD))	9.7 (0.8)	-	9.7 (0.6)	1 (0.4)	0.73
Apgar 3 (mean (SD))	9.9 (0.5)	-	9.9 (0.4)	1 (0.4)	0.86
Neonatal Complications	2.8%	-	6.6%	-	0.07

* caesarean section excluded

## Discussion

Compared to women who are legal residents of Geneva, undocumented migrants had more unintended pregnancies, use preventive measures less frequently, delayed prenatal care more, and were exposed to more violence during pregnancy.

Delayed use of prenatal care remains problematic among undocumented migrants in Geneva: the first pregnancy visit occurred more than 4 weeks later than for women with a legal residence permit, and prenatal care began during the first trimester in only 63% of the undocumented compared to 96% of controls. Similar difficulties were observed for undocumented pregnant migrants in Colorado, US [10]. The United Nations has indicated that one high priority "Millennium goal" is to improve maternal health throughout the world [11]. Even if the existence of a free health care unit facilitates access to care, there is clearly a need to find ways to improve use of care and particularly early pregnancy care for undocumented women. In our experience, the cost of health care is a major barrier, particularly in countries like Switzerland where each individual has to arrange and pay for their own health insurance and where over 90% of the undocumented migrants lack health insurance. Improved health care access for undocumented migrants requires creative financial solutions, including being free or of minimal charge, but also language competencies of health care providers and administrative staff. Furthermore, protection has to be guaranteed: undocumented migrants would hardly be likely to contact a health care provider if they feared potential notification of their stay to the police and any other subsequent legal sequelae.

Undocumented pregnant migrants in Geneva were mostly young and single Latin-American women of whom an important percentage lacked social and emotional support. They were living in poor housing conditions and one in five of them had no or only an occasional relationship with the father of their child.

Despite our findings that prenatal care was delayed and preterm births were more frequent in undocumented migrants (9%vs.4%, p = 0.09), health outcomes such as complications during pregnancy, delivery, and post-partum were similar in both groups, and neonatal outcomes even tended to be slightly better in the undocumented. These relatively good health outcomes might be explained by a selection of the fittest women during migration, which has been conceptualized under the "healthy migrant effect" [6,12]. Alternatively, it could be hypothesized that good birth outcomes might be explained by the fact that women who were lost to follow-up might have had worse risks. Nonetheless, when comparing undocumented women who delivered to those who left the country, no particular risk profile could be identified.

Considering drugs and alcohol abuse, undocumented pregnant migrants showed a healthier pattern than control women. Prenatal alcohol exposure is a major cause of foetal defects and neurodevelopmental problems and the most frequent cause of avoidable mental retardation [13]. In our population, most women stopped their alcohol intake with the onset of pregnancy. Nevertheless, 30% of the control group and 12% of undocumented migrants consumed alcohol during pregnancy with a notable proportion of binge drinking (16% versus 6%). Healthcare professionals must be aware of this major problem.

Seroprevalences in undocumented women corresponded to their countries of origin, mainly Latin-America. They were better immunized against Toxoplasma gondii and CMV but less so against rubella than controls. Toxoplasma immunity prevalence among controls was 42%, similar to what has been found for the Swiss general population (46%) [14], whereas the Toxoplasma immunity prevalence in Latin-America (67%) is similar to that of the undocumented migrants in this study. Seroprevalence of rubella is known to vary across countries, with lower rates in Latin-America [15,16], where congenital rubella syndrome is an under-recognized public health problem [17].

The high prevalence of unintended pregnancies among undocumented migrants (75%) highlights an important public health issue and confirms our previous study where we found a similar rate among undocumented migrants in Geneva [7]. In contrast, the control group reported only 20% of unintended pregnancies. To our knowledge, this is the first time that unintended pregnancies resulting in live births have been studied for women *with* a legal residency permit in Switzerland. International comparisons show large differences between and uncertainties within countries, which indicates the complexity of measurement of unintended pregnancies [18,19]: 10 to 31% in Great-Britain [20,21], 16 to 20% in France [22], and up to 55% in Colombia resulting in live births [23]. In the US 49% of all pregnancies are estimated to be unintended [24], of which 33% to 49% result in live births, with large differences between the states [25,26]. Known factors associated with unintendedness [23,27,28], such as delayed prenatal care, not being married, or exposure to violence were also observed in our study.

Exposure to violence has frequently been reported, particularly among women with unintended pregnancies and during pregnancy, as was the case for 11% of the undocumented migrants in our study [23,29]. Consequently, it is important to ask pregnant women systematically and repeatedly about violence exposure [30]. It was unexpected that only 1.3% of the controls reported being exposed to violence during pregnancy, which contrasts with 7% found in a survey conducted 10 years before at the same hospital [31]. The latter study investigated violence prevalence as a major outcome, which could have influenced the women's responses and explain the higher prevalence.

Seventy-nine percent of the women with unintended pregnancies did not use any (48%) or used unreliable (31%) contraceptive measures, and 61% were unaware of emergency contraception (Levonorgestrel) which can prevent pregnancy up to 72 hours after intercourse and can be obtained without medical prescription in Geneva [32,33]. The important difficulties concerning knowledge, access, and use of preventive measures are also illustrated by the under-utilisation of cervical smear (Pap) tests and breast examination. Pap test under-use corresponds to the well-known lack of lifetime screening in many parts in Latin-America and underlines the need for language- and culturally-appropriate education [34,35].

The relationship between residency permit and the main outcomes might be influenced by age, origin, civil status, education, duration of residence in Geneva, and having emotional support and/or a family member in Geneva. Using multiple logistic regression analysis we found that civil status was an important confounder of the relationship between residency status and three main outcomes: unintendedness of pregnancy, delayed prenatal care, and less use of Pap tests by undocumented migrants. Other potential confounders had no significant influence on the main outcome in our study and were therefore not included in the adjusted analyses.

Our study confirms the close relationship between illegality and poverty. Undocumented migrants earned 13 SFr per hour (≈8 Euro) which is 40% lower than the minimal mandatory hourly wage in Geneva. Furthermore, undocumented migrant status is associated with isolation, stigma, and fear. Further research is needed to better elucidate these complex influences in order to implement effective programmatic solutions for the main problems pointed out here.

The present study has several strengths. First, to the best of our knowledge, that pregnancy characteristics have been studied in undocumented migrants and compared to a local control group is unique in Europe. Second, we prospectively included a relatively large systematic sample from this hard-to-reach population. Third, we investigated for the first time unintended pregnancies resulting in live births for women with a legal residency permit in Switzerland.

Some limitations of the present study include: First, fifty-three undocumented women (33%) left Geneva and were considered as lost to follow-up but their baseline characteristics did not differ from women who delivered at the women's hospital. Socio-demographic variables were missing for up to 24% of the study participants. On the other hand, except for knowledge of the emergency contraceptive pill, other data on preventive measures, histories of voluntary pregnancy termination and violence, as well as blood tests were missing for less than 10% of the study participants. Second, the sample size limits the power of the study. Third, the time of data collection was not identical for both study groups: first to third trimester for undocumented migrants vs. the last trimester for controls. This difference might be neglectable for the large majority of the questions; nevertheless it might have influenced responses concerning intendedness of pregnancy. However, when comparing unintendedness by trimester, no significant difference was found for undocumented women. Fourth, potential reporting bias has to be considered, as the study group was not blinded and midwives might have been more probing to detect daily-life difficulties among undocumented migrants. However, the two midwives who administered the questionnaire provided the same quality and frequency of clinical follow-up to undocumented migrants as to insured women with a residency permit and they were trained to administer the questionnaires in a precise and neutral way similarly for both groups. Fifth, undocumented women might not be representative of the total undocumented population of Geneva. However, several aspects do lead us to believe that this study reached a substantial proportion of pregnant, undocumented women in Geneva and is therefore representative of them: 1) The free medical care unit is well known by this hard-to-reach population; 2) The proportion of Latin-Americans (84%) is similar to that found by other sources: for example, in investigating the origins of undocumented workers, the Geneva trade union recently found 76% were Latin-Americans [36]; 3) In order to achieve optimal participation, undocumented women were enrolled in collaboration with the woman's hospital, which is the only public obstetrical hospital in Geneva and the only place where uninsured and undocumented women can deliver at low or no cost. Finally, it might still be possible that the study sample still differs from the whole undocumented population of Geneva, which is unknown by definition and thus not officially enumerated. Although our control group was not a random sample from the general population, it was obtained by systematically sampling all the women with valid residence permits who were seen on selected days at the same hospital by the same midwife during the same time period that the sample of undocumented migrants was obtained.

## Conclusion

Compared to women who are legal residents of Geneva, undocumented migrants have more unintended pregnancies and delayed prenatal care, use fewer preventive measures and are exposed to more violence during pregnancy. Not having a legal residency permit therefore suggests a particular vulnerability for pregnant women. This study underscores the need for better access to prenatal care and routine screening for violence exposure during pregnancy for undocumented migrants. Furthermore, health care systems should provide language- and culturally-appropriate education on contraception, family planning and cervical cancer screening.

## Abbreviations

CI: 95% confidence interval; CHF: Swiss francs; OR: odds ratio; SD: standard deviation; y: years.

## Competing interests

The author(s) declare that they have no competing interests.

## Authors' contributions

HW conceived of the study, participated in its design and coordination, performed the statistical analysis, interpretation of the data and drafted the manuscript, ME participated in the study design, coordination, interpretation of the data and gave critical contribution to the manuscript, APL participated in the study design, coordination and interpretation of the data, MCC gave critical contribution to the manuscript, JDM and NA contributed substantially to the acquisition of data, JBD and JMG gave critical contribution to the manuscript and OI participated in the study design and coordination and gave critical contribution to the manuscript. All authors read and approved the final manuscript.

## Pre-publication history

The pre-publication history for this paper can be accessed here:


